# Supplementary figures and images for: Energy consumption prediction using the GRU-MMattention-LightGBM model with features of Prophet decomposition
Source: PLoS One. 2023 Jan 17;18(1):e0277085. doi: 10.1371/journal.pone.0277085 (PMC9844920; doi:10.1371/journal.pone.0277085)

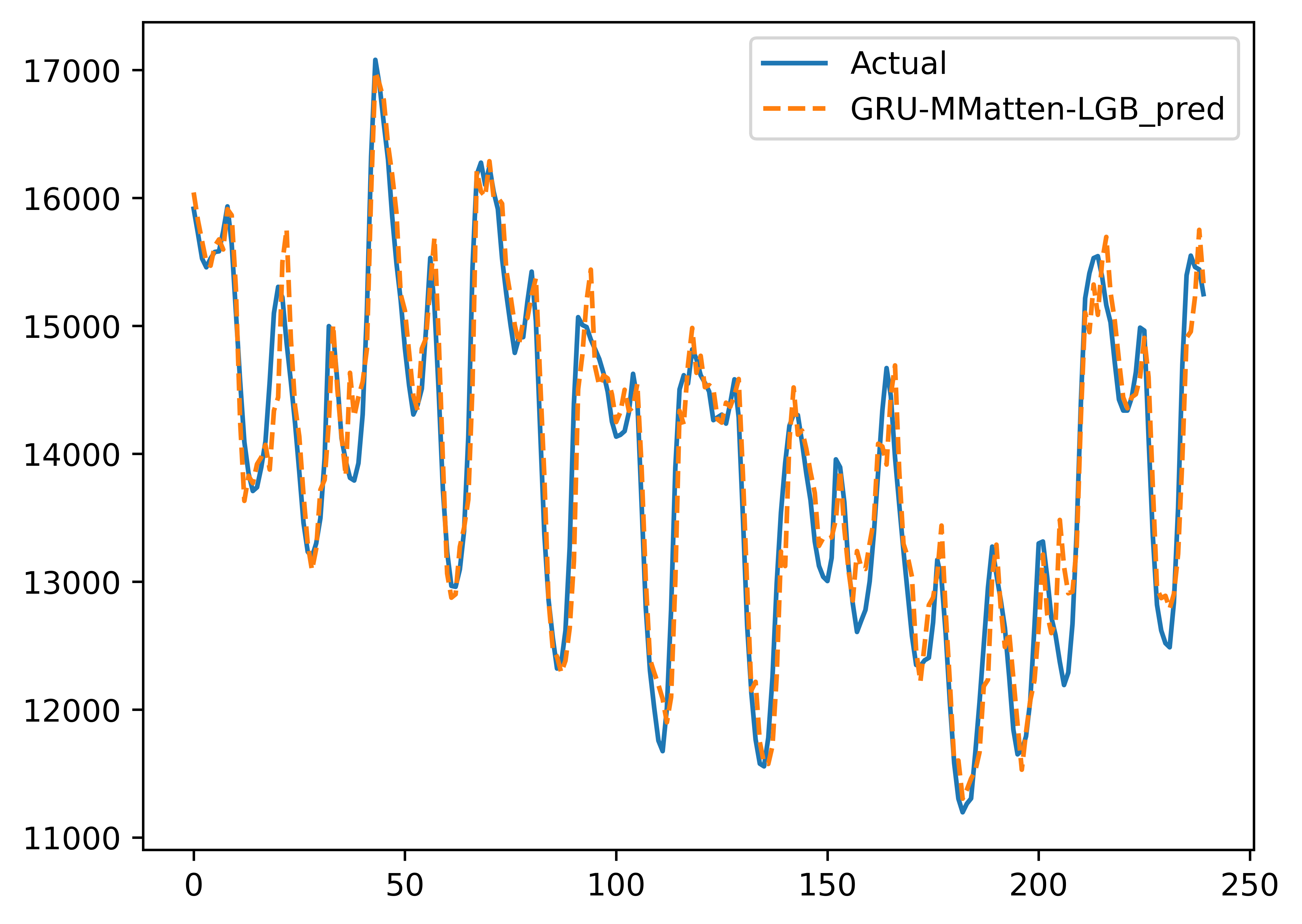

Supplement: S1 Data — (ZIP) [file pone.0277085.s001.zip › ElectricityPrediction/GRU-MMatten-LGB.png]

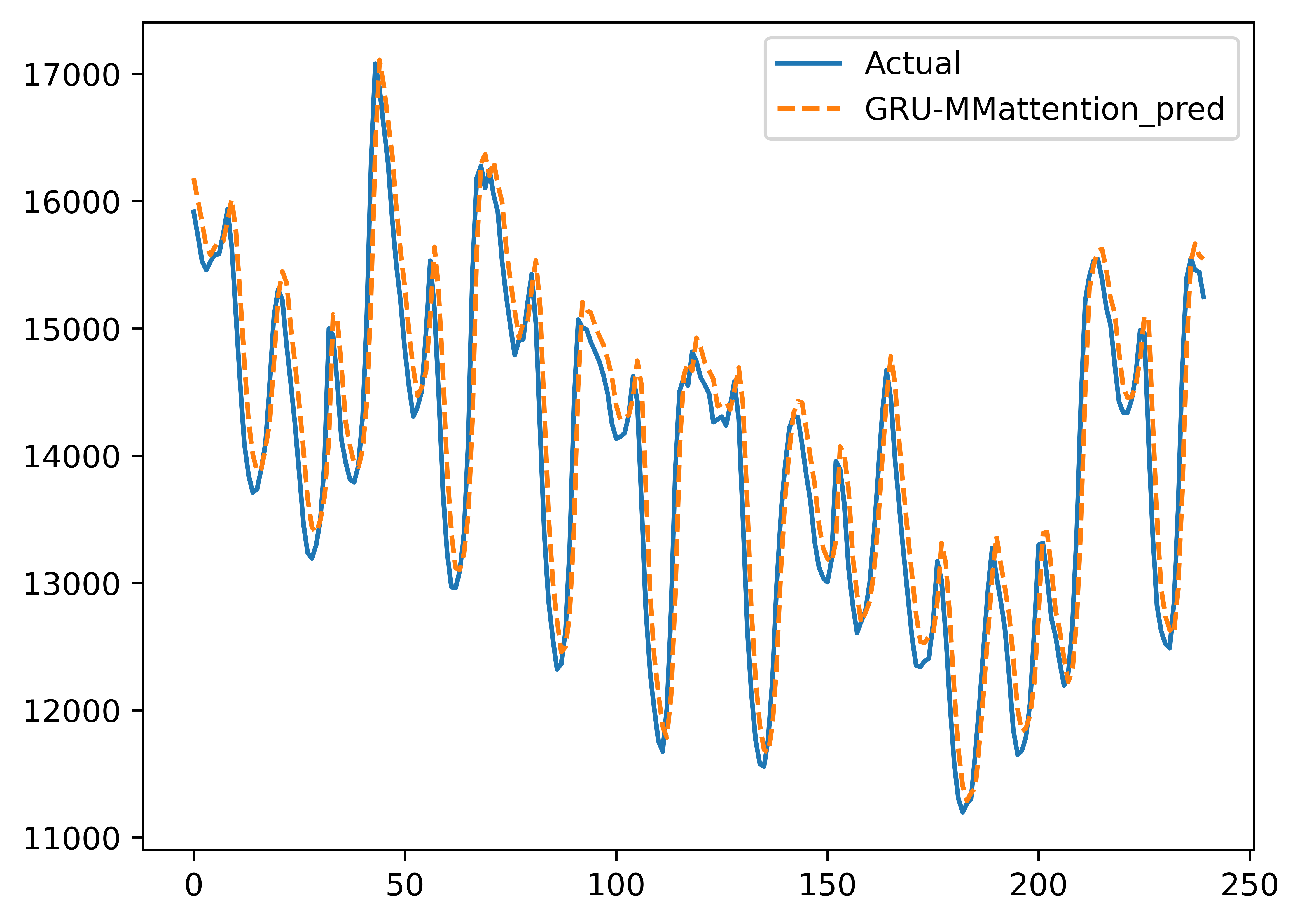

Supplement: S1 Data — (ZIP) [file pone.0277085.s001.zip › ElectricityPrediction/GRU-MMattention.png]

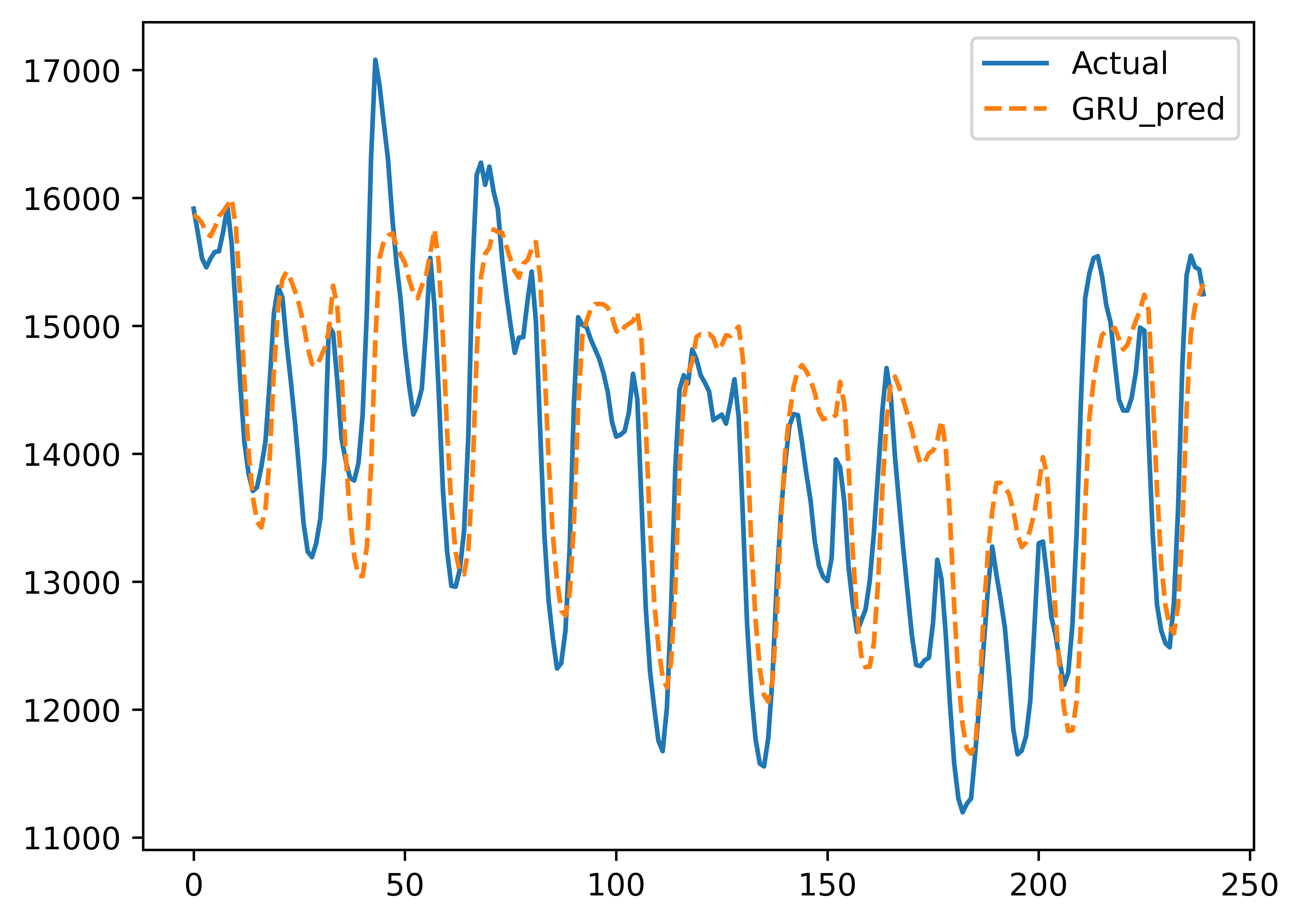

Supplement: S1 Data — (ZIP) [file pone.0277085.s001.zip › ElectricityPrediction/GRU.png]

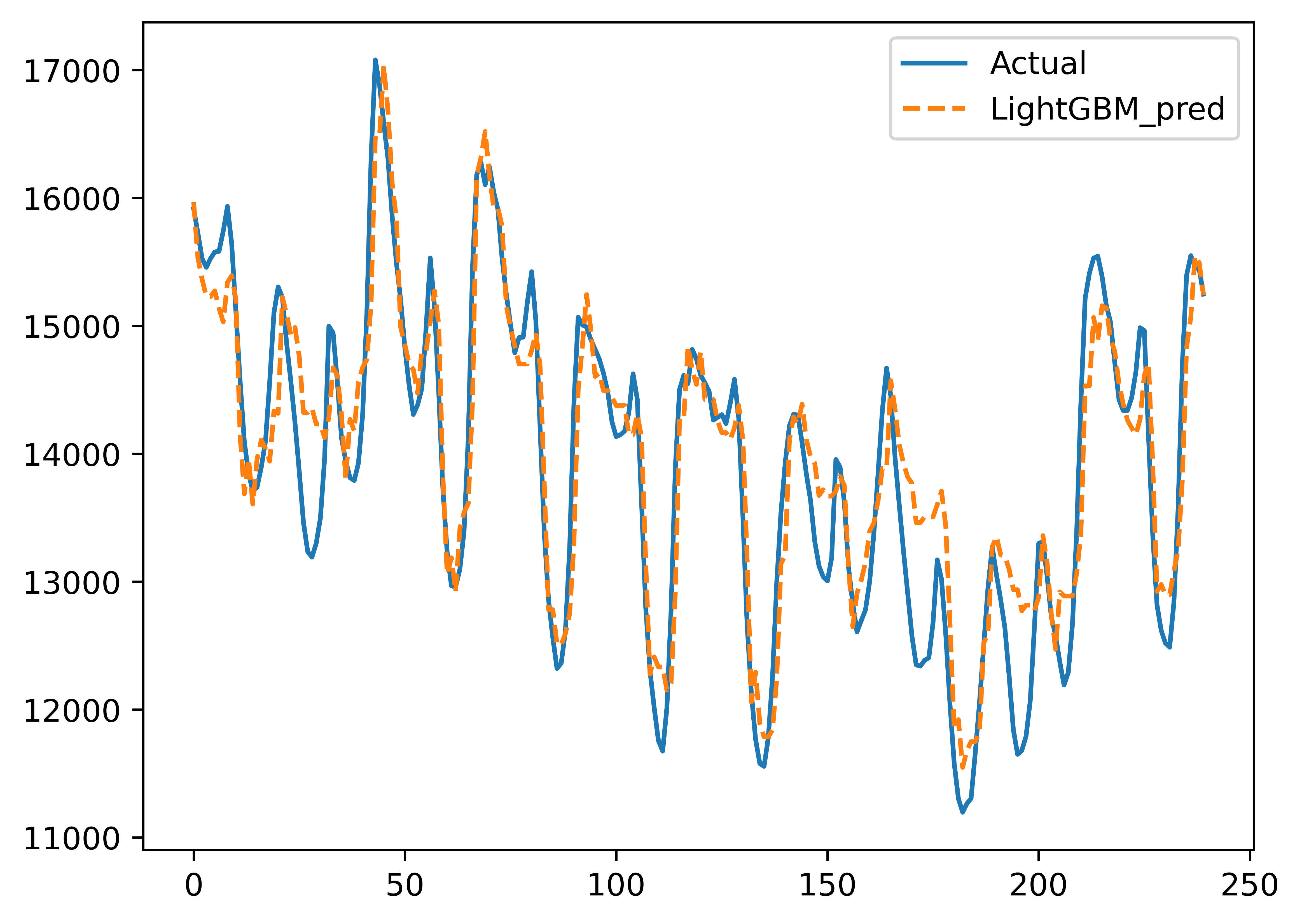

Supplement: S1 Data — (ZIP) [file pone.0277085.s001.zip › ElectricityPrediction/LightGBM.png]
