# Supplementary material for: Energy consumption prediction using the GRU-MMattention-LightGBM model with features of Prophet decomposition
Source: PLoS One. 2023 Jan 17;18(1):e0277085. doi: 10.1371/journal.pone.0277085 (PMC9844920; doi:10.1371/journal.pone.0277085)
Supplement: S1 Data — (ZIP) [file pone.0277085.s001.zip › ElectricityPrediction/Document description.docx]

# config.py

保存数据读取、模型训练等阶段的超参数

Save the hyperparameters of data reading, model training, etc

# main.py

project的入口。调用run_models.py中的函数对GRU, GRU-MMattention, GRU-MMattention-LGB 模型训练并测试，

Project entry. Call the function in run_models.py to train and test GRU, GrU-Mmat-LGB model,

# ckpt

The checkpoint file of the model is saved in this folder

Checkpointing is implemented by rerunning a forward-pass segment for each checkpointed segment during backward. This can cause persistent states like the RNG state to be advanced than they would without checkpointing.

The ckpt folder naming rule is : ModelName-EpochNumbers-StepNumbers-MSE of the validation dataset

LightGBM.joblib保存了基于电力数据来训练的LightGBM模型

LightGBM. Joblib keeps the LightGBM model trained based on power data

# data

RawData includes power consumption data

Electricity contains energy consumption data of the US PJM regional energy supply company

## data_cleaned_prophet_minmax_2D

经过prophet特征提取且归一化处理后的电力能耗预测数据，按照lightGBM模型规范要求保存为2D的array

The predicted data of energy consumption after prophet feature extraction and normalized processing are saved as 2D array according to the requirements of lightGBM model specifications

## data_cleaned_prophet_minmax

经过prophet特征提取且归一化处理后的电力能耗预测数据，按照torch格式要求保存为3D的array

The predicted data of energy consumption after prophet feature extraction and normalized processing are saved as 3D array according to the requirements of lightGBM model specifications

**lag_j=3**

按照滚动时间窗口进行特征提取且归一化处理后的电力能耗预测数据，窗口长度为3，按照torch格式要求保存为3D的array

The energy consumption data after feature extraction and normalized processing are processed according to the rolling time window with a window length of 3. The data is saved as a 3D array according to the torch format

**lag_j=3_2D**

时间窗口滚动特征提取且归一化处理后的电力能耗预测数据，窗口长度为3，按照lightGBM模型规范要求保存为2D的array

The energy consumption prediction data after the time window rolling feature extraction and normalized processing, with window length of 3, are saved as 2D array according to the requirements of lightGBM model specifications

# Datasets：

elec_dataset.py

按照pytroch的风格要求将导入的数据抽象为类

Abstract the imported data into classes as required by Pytroch's style

# final_ckpy:

Checkpoint files of GRU-mmatten-LGB（combined model）

# lightning_logs:

模型运行过程中的日志文件，用于查询错误。

Log files during the model run to check for errors.

# models:

**combination.py**

define GRU-MMattention-LGB method.

**GRU_atten.py**

define GRU-MMattention method。

**normal.py**

define GRU method。

**run_models.py**

分别定义训练并测试普通GRU模型、浅层模型（LGB等）、GRU-atten模型的函数。

The functions of training and testing ordinary GRU model, shallow model (LGB, etc.) and GRU-ATTEN model are defined respectively.

# utils:

**utils.py**

数据导入函数、反归一化函数。

Data import method, inverse normalization method.

**1.How to run this project?**

**（1）Get results of the original experiment without noise**

set the parameter ‘noise=False’ in line 18 of the ‘main.py’

then run ‘main.py’

**（2）Get results of the noisy experiment**

set the parameter ‘noise=True’ in line 18 of the ‘main.py’

then run ‘main.py’

**2.How to specify one model to run for the results?**

As the code shown in the ‘**main.py**’,all the methods are set ‘True’ for running to the results.

For instance:

If a GRU method is not expected to be shown, you can set the parameter’ gru = False’.The module of GRU will not be activate in the process.

If a LightGBM method is not expected to be shown, you can set the parameter’ lgb = False’. The module of LightGBM will not be activate in the process.

If a GRU-MMattention method is not expected to be shown, you can set the parameter’ gru_atten = False’. The module of LightGBM will not be activate in the process.

If a GRU-MMattention-LGB method is not expected to be shown, you can set the parameter’ combined_model = False’. The module of GRU-MMattention-LGB will not be activate in the process.

**3. How to specify LightGBM model to run for the results of noisy experiment?**

Because machine learning lightGBM uses a different library form deep learning methods, it is impossible to reset the random seeds of all models at the same time. We are only able to reset the random seed of GRU, GRU-MMattention, GRU-MMattention-LGB at the same time and make it consistent. So to get lightGBM's 10 results under noisy conditions, you have to manually adjust the random seed each time you run it.

**4.How to test the results of the noisy experiment?**

We need to use stats library in **SCIPY**. First, import GRU, GRU-MMattention, GRU-MMattention-LGB results from noise experiment into dataframe, and then use **stats.shapiro** method to check the normality of the results. Finally, **stats.ttest_1samp** is used to test the mean value of the noise experiment results. Here, the mean value of each method is set as the MAPE of the original experiment results without noise.
